# Development and implementation of a value framework for rapid health technology assessment reports: enhancing evidence-informed decision making in resource-constrained settings

**DOI:** 10.1017/S0266462325100160

**Published:** 2025-07-21

**Authors:** Andrea Alcaraz, Fernando Argento, Veronica Alfie, Sebastián García Martí, Ariel Bardach, Agustín Ciapponi, Federico Augustovski, Andres Pichon-Riviere

**Affiliations:** 1 Health Technology Assessment and Economic Evaluation Department, Institute for Clinical Effectiveness and Health Policy, Buenos Aires, Argentina; 2 Centro de Investigación en Evaluación de Tecnologías Sanitarias y Políticas de Salud (CIESP), National Scientific and Technical Research Council (CONICET), Buenos Aires, Argentina

**Keywords:** health technology assessment, evidence-based practice, developing countries, health policy, rapid assessment, value frameworks

## Abstract

**Objectives:**

Value frameworks play a crucial role in bridging the gap between evidence and decision making in health care, particularly in settings with limited resources as low- and middle-income countries (LMIC). In this study, we present the development of a value framework (VF) targeted to provide coverage recommendations in rapid health technology assessment reports (rHTA) as well as its first 5 years of implementation.

**Methods:**

We performed an exhaustive literature search with the aim to identify existing VFs and their dimensions followed by the generation of a VF proposal through a mixed methods, qualitative–quantitative approach including a Delphi panel to weigh the criteria and correlate them with the subsequent recommendations. To describe its implementation, we present the results of 264 rHTA reports from 2017 to 2022.

**Results:**

The value framework has three main domains (quality of evidence, net benefit, and economic impact). We adapted widely used methodologies for quality of evidence and net benefit domains. The economic impact domain was the most complex to assess, so an ad hoc method was developed. Analysis of 265 HTAs revealed the distribution of recommendations across different criteria and technology types. Most were for drugs (40.5 percent) or therapeutic procedures (36 percent). With a five-category final recommendation, 0.8 percent were favorable, 19.7 percent were uncertain, and 44 percent were unfavorable.

**Conclusion:**

The VF demonstrated its versatility and practicality in meeting the needs of rHTA audience, and can facilitate evidence-informed decision making. This VF serves as a valuable tool for conducting adaptive rHTAs and supports decision-making processes in Argentina and similar LMIC contexts.

## What is already known on this topic

Prior to this study, it was known that value frameworks play a significant role in bridging the gap between evidence and decision making in health care, especially in environments with limited financial resources. However, there was a need for a tailored value framework specifically for rapid health technology assessments (rHTAs) to enhance the quality and efficiency of coverage recommendations.

## What this study adds

This study developed and implemented a value framework (VF) for use in rHTAs and demonstrated its practicality and versatility. The framework incorporated adapted methodologies for quality of evidence, net benefit, and economic impact domains. The study provided detailed insights into the distribution of recommendations across different criteria and technology types, revealing that most recommendations were for drugs or therapeutic procedures, with a significant portion being unfavorable or uncertain.

## How this study might affect research, practice, or policy

The implications of this study are significant for the practice of rHTAs in Argentina and similar contexts. The developed VF supports more informed and evidence-based decision-making processes, which can lead to better resource allocation and healthcare outcomes. This framework can be adapted and utilized by other regions and countries, potentially standardizing the approach to rapid health technology assessments globally.

## Introduction

Value assessments act as a bridge between evidence and decision making. They can be used to assess drugs and other healthcare technologies to enable the incorporation of effective interventions into the health system. Value frameworks bring together different decision-making domains, making the process more systematic and transparent, which is particularly important when budgets are limited (1).

Health technologies are defined as medicines, tests, devices, vaccines, procedures, programs, or systems, and with their increasing use over the years, they have become an important part of a country’s healthcare system (2). However, healthcare policymakers must constantly make decisions regarding which new interventions should be financially covered, who will pay for them, and how they will be delivered to patients. They seek to obtain the best health outcomes through the most efficient use of available resources (3). As low- and middle-income countries (LMICs) strive to achieve universal health care, they are confronted with budget constraints. In such situations, it is critical to provide robust, trustworthy, and detailed information that allows decision makers to arrive at evidence-informed decisions regarding priorities (3).

Health technology assessment (HTA) is a process for comprehensively evaluating health technology’s value and potential impact within a healthcare system. The assessment process is based on the use of criteria to help define the value of technologies. Value frameworks define the included criteria to help make the process of evaluating a new technology more transparent for decision making (4).

Value frameworks help structure the assessment of health technologies by specifying a set of criteria that define and operationalize their value. These criteria – such as clinical benefit, quality of evidence, and economic impact – must be conceptually relevant, measurable, and aligned with decision-making needs. Therefore, a value framework is needed that aligns the criteria with both the conceptual definition of value and its practical measurement, ensuring consistency and relevance across evaluations.

Since 2002, the HTA department of the Institute for Clinical Effectiveness and Health Policy (IECS) has supported the decision-making processes of the national Ministry of Health, provincial health authorities, social security institutions, and the private sector in Argentina. This support is provided through the production of rapid health technology assessment reports (rapid HTAs), which are streamlined evaluations conducted in a short time frame (typically 4–8 weeks) using existing evidence and adapted methodologies to meet urgent decision-making needs. These assessments are carried out in response to formal requests from the aforementioned institutions, which act as both funders and end-users of the evaluations.

In recent years, adaptive health technology assessment (aHTA) has emerged as a pragmatic approach to address growing demands for timely decision making, particularly in low- and middle-income countries (LMICs), including Argentina. IECS has incorporated aHTA strategies to meet these demands under constraints such as limited data availability, urgency, and restricted budgets. However, existing aHTA methodologies are often criticized for lacking transparency and offering limited guidance on how to synthesize multidimensional evidence to support decisions.

As part of an initiative to improve the methodology for conducting evaluations and to generate a coverage recommendation that was transparent and easy to interpret, in 2017, we set out to define a value framework (VF) for issuing recommendations. This framework offers decision makers a simplified overview of recommendations while simultaneously ensuring increased transparency concerning its underlying values.

This article aims to present the VF and its visual tool developed by IECS for undertaking rapid HTA and describe our agency experiences after almost 5 years of implementation.

## Methods

### Development of the value framework

The first step in developing the VF in 2016 was to perform a targeted literature review to identify existing value frameworks (VFs) and their key dimensions. Although this process was not systematic, it focused on widely used frameworks from leading HTA agencies. From this review, the most frequently included domains were identified and compiled. A group of nine researchers with extensive experience in HTA selected those domains that were both recurrent in the literature and feasible to assess in the context of rapid evaluations. For each selected domain, the team identified available tools; where no suitable tool existed, they developed ad hoc methods. This process was later validated by a panel of 12 HTA researchers from IECS.

A matrix was then constructed to translate domain-specific assessments (each one with three to four categories) into five summary levels of coverage recommendation. These levels ranged from fully in favor of coverage to completely against it and were based on the performance of the technology across the three evaluated domains. To define this linkage between domain-level assessments and the overall recommendation, a Delphi panel was conducted with nine senior researchers from IECS. The Delphi process consisted of two rounds. In the first round, each researcher assigned a recommendation level – fully in favor (green), moderately in favor (yellow-green), uncertain (yellow), moderately against (yellow-red), or strongly against (red) – to each possible combination of domain scores. A second round was conducted to resolve any disagreements and reach a consensus.

Once the process was completed, the matrix of overall recommendations undertook a face-validity check through interviews with eight decision makers from the Superintendency of Health Services and public and private health funders. The VF and its recommendation matrix were piloted through the completion of 24 rapid HTAs to evaluate its implementability and to establish possible improvements (5).

### Value framework implementation

The value framework (VF) was implemented within the rapid HTA process in response to requests from public and private health institutions. Each assessment was completed within 4 to 8 weeks and followed a standardized process. Reports began with a scoping phase to define the research question in PICO format and identify relevant stakeholders for each technology. Two IECS researchers led the report preparation, with support from subject matter experts as needed and input from a multidisciplinary team of 20–25 professionals – including epidemiologists, clinicians, health economists, and other health professionals with HTA training.

Structured deliberation meetings were held at predefined points in the process to reach a consensus on the classification of each domain within the VF. These discussions focused on the technology’s performance in terms of clinical benefit, quality of evidence, and economic impact. Although equity and rare disease status were not formal domains within the framework, they were considered through deliberative judgment when contextually relevant.

After completion, reports underwent an embargo period and a public consultation phase, during which feedback from manufacturers, professional societies, funders, and patient organizations could lead to revisions before publication. In cases where a technology received more than one recommendation, this was due to a broad PICO question covering different subpopulations or comparators.

To evaluate the implementation of the VF, we reviewed all HTAs conducted using the framework over a 5-year period. This included analyzing how each domain was classified, the type of technology and health condition assessed, the frequency and timing of report requests, and the overall production timelines. We recorded the time from the receipt of the request to the project assignment, and from there, to report publication on the IECS website, including the finalization, embargo, and translation phases. When available, we also analyzed the outcomes according to the domain classifications and final recommendations issued.

## Results

### Development of the value framework

A focused literature search conducted in 2017 identified value frameworks developed by institutions such as the *National Institute for Health and Care Excellence* (NICE) in the United Kingdom, the *Canadian Agency for Drugs and Technologies in Health* (CADTH), the *Institute for Quality and Efficiency in Health Care* (IQWiG) in Germany, the *American Society of Clinical Oncology* (ASCO), and the *American College of Cardiolog*y (ACC) and *European Society of Cardiology* (ESC) (6–10). Based on this analysis, the initial criteria considered for inclusion in the IECS value framework were efficacy, safety, quality of evidence, and economic impact (including cost-effectiveness and budget impact). Efficacy and safety were later combined into a single “net benefit” domain to simplify the assessment while preserving content validity. Other aspects such as equity or rare disease status were acknowledged as relevant, but were excluded from the core framework due to operational challenges and limited data availability. However, these aspects could be discussed during the deliberative process when contextually important.

Each of the three selected domains was adapted for use in rapid HTAs. For evidence quality, the *Grading of Recommendations Assessment, Development, and Evaluation* (GRADE) approach was used due to its transparency and broad acceptance, but it was adapted to allow a global judgment of evidence across outcomes rather than outcome-by-outcome grading. The quality was anchored in the most critical outcome(s) defined in the PICO question, and adjusted through deliberation if other outcomes significantly changed the certainty of the body of evidence. The categories used were high, moderate, low, and very low/null (11). For the net benefit domain, methods were adapted from the IQWiG framework (10), using the most important outcome as an anchor and deliberating whether other outcomes or harms modified the overall assessment. The resulting categories were major, considerable, minor, and marginal/null/uncertain/negative.

No existing method for assessing cost-effectiveness or budget impact was considered fully applicable in this context. Standard approaches based on cost-effectiveness ratios and predefined thresholds were not feasible in Argentina due to the lack of local thresholds and economic evaluations, as well as the short timeframe of rapid assessments. To address this, a group of seven senior IECS researchers developed a context-adapted method drawing on international definitions, budget impact criteria from countries such as Chile and England (adjusted for local purchasing power), and evidence extrapolation strategies. When available, local data were incorporated directly. Otherwise, estimations were informed by international sources. The assessment considered incremental cost versus comparator, population size, net benefit, and prior coverage decisions in other systems, and classified economic impact as favorable, uncertain, or unfavorable.

**Figure 1.**
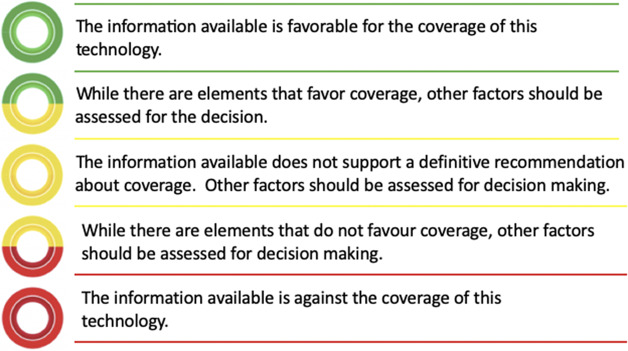
Possible recommendations for the coverage decision.

These three domains were combined in a recommendation matrix (Annex 1), which produced a five-level color-coded recommendation scale – from clearly in favor to clearly against coverage – with intermediate categories suggesting the need for further evidence (Figure 1). During pilot testing, no major difficulties were reported, though the labels of the economic domain were refined for clarity (Table 1). When additional contextual factors arose – such as equity considerations – a deliberative process allowed the research team to shift the recommendation up or down by one level to better reflect the overall value of the technology.

**Table 1.** Value criteria of different domains

Net benefit	
*Major*	▪ Survival (RR ≤0.85) or ▪ Serious symptoms (or severe or late complications) and serious adverse events/quality of life (RR ≤0.75)
*Considerable*	▪ Survival (RR >0.85 and ≤0.95) ▪ Serious symptoms (or severe or late complications) and serious adverse events/quality of life (RR >0.75 and ≤0.90) ▪ Nonserious symptoms (or not severe or late complications) and nonserious adverse events/quality of life (RR ≤0.80)
*Minor*	▪ Survival (RR >0.95 and <1) ▪ Serious symptoms (or severe or late complications) and serious adverse events/quality of life (RR >0.90 and <1) ▪ Nonserious symptoms (or not severe or late complications) and nonserious adverse events (RR >0.80 and ≤0.90) ▪ Other relevant benefits for the patient or the health system (e.g., simple administration, treatment convenience, treatment duration, less organizational impact, fewer hospitalization days)
*Marginal/null/uncertain/negative*	▪ The benefits are nonsignificant or there is no benefit, or it is uncertain, or it is harmful
Economic aspects (cost-effectiveness and budget impact)	
*Favorable*	▪ There is adequate quality of evidence, showing cost savings in Argentina, or ▪ There is adequate quality evidence, showing that it is cost-effective* in Argentina, and that it does not represent a high budgetary impact^§^, or It simultaneously meets the following four conditions: (1) the incremental cost versus the comparator is not high^¥^, (2) the population involved is small^£^, (3) the size of net benefit relative to cost suggests possible be cost-effectiveness, and (4) it does not represent a high budgetary impact^§^
*Uncertain*	It does not meet the criteria for *favorable* or *unfavorable* (this includes cost-effective interventions with high budgetary impact)
*Not favorable*	▪ There is adequate evidence quality, showing that it is cost-effective in Argentina, or Although there is no adequate quality of evidence on cost-effectiveness in Argentina, there are enough elements to consider that it would NOT be cost-effective (e.g., the cost is high in relation to the comparator and the relation between the cost of the intervention and the size of net benefit is clearly unfavorable – e.g., more than 3 gross domestic product [GDP] per year of life or the QALY [quality-adjusted life-year]; there is evidence that it is not cost-effective in other countries; or it was explicitly excluded from coverage of other health systems because of their budgetary impact and/or lack of cost-effectiveness)
Quality of evidence	
*High*	It is very unlikely that future research changes the effect estimate
*Moderate*	It is likely that future research may change the effect estimate
*Low*	It is very likely that future research changes the effect estimate
*Very low/null*	Any effect estimation is uncertain

RR = relative risk; *cost-effectiveness*:* a technology is considered to be cost-effective for Argentina if there are studies carried out for the country considered to be of good quality that estimate values equal to or less than 1 gross domestic product (GDP) per capita per year gained or QALY (2 GDP in case of rare diseases);

^§^high budget impact: the expected annual budget impact of incorporating the technology is greater than 15 annual health expenditures per capita per 100,000 people (representing an increase in health expenditure of more than 0.015 percent); ^¥^high incremental cost versus comparator: greater than annual per capita health expenditure in Argentina, or greater than 25 percent of the annual amount that is considered catastrophic for a household (according to the WHO definition, which considers catastrophic expenditure to be greater than 40 percent of nonbasic household expenses); *^£^*small affected population: up to 15 cases for every 100,000 inhabitants.

### Value framework implementation

For the period from May 2017 to May 2022, 264 HTAs were completed, of which 166 included one recommendation, 87 included two, and 11 included more than three, totaling 375 recommendations (as some reports could include more than one recommendation depending on the subpopulations or comparators considered). The time between the request for assessment and its assignment to a researcher was a median of 4 days (IQR: 2–10). The time from assignment to publication on the web page was 42 days (IQR: 33.25–52.75), which included the preparation of the final report, the embargo period, and translation.

Most of the assessments focused on drugs (40.3 percent) or therapeutic procedures (35.7 percent). The most prevalent clinical areas assessed were cancer (16.6 percent) and neurology (15.8 percent) (Table 2).

**Table 2.** Characteristics of the completed HTAs and recommendations

Variable	Characteristic	Value *N* (%)
Technology type	Drug Therapeutic procedure Diagnostic Device	107 (40.5) 95 (36) 38 (14.4) 24 (9.1)
Healthcare area	Cancer Neurology Musculoskeletal Endocrinology and metabolism Eye Cardiovascular Infectious diseases Others Respiratory system Urinary and kidney disorders Gastrointestinal Skin Women’s health Ear, nose, and throat Accidents and trauma Covid–19 Pregnancy and childbirth Nutrition and obesity Mental health Children’s health Sexual and reproductive health Hematology	43 (16.3) 42 (15.9) 26 (9.8) 19 (7.2) 16 (6.1) 15 (5.7) 14 (5.3) 13 (4.9) 12 (4.5) 12 (4.5) 11 (4.2) 7 (2.7) 7 (2.7) 6 (2.3) 4 (1.5) 3 (1.1) 3 (1.1) 3 (1.1) 3 (1.1) 2 (0.8) 2 (0.8) 1 (0.4)
Recommendation	Totally favorable (green) Moderately favorable (yellow-green) Uncertain (yellow) Moderately unfavorable (yellow-red) Totally unfavorable (red)	3 (0.8) 49 (13.1) 74 (19.7) 83 (22.1) 166 (44.3)

**Figure 2.**
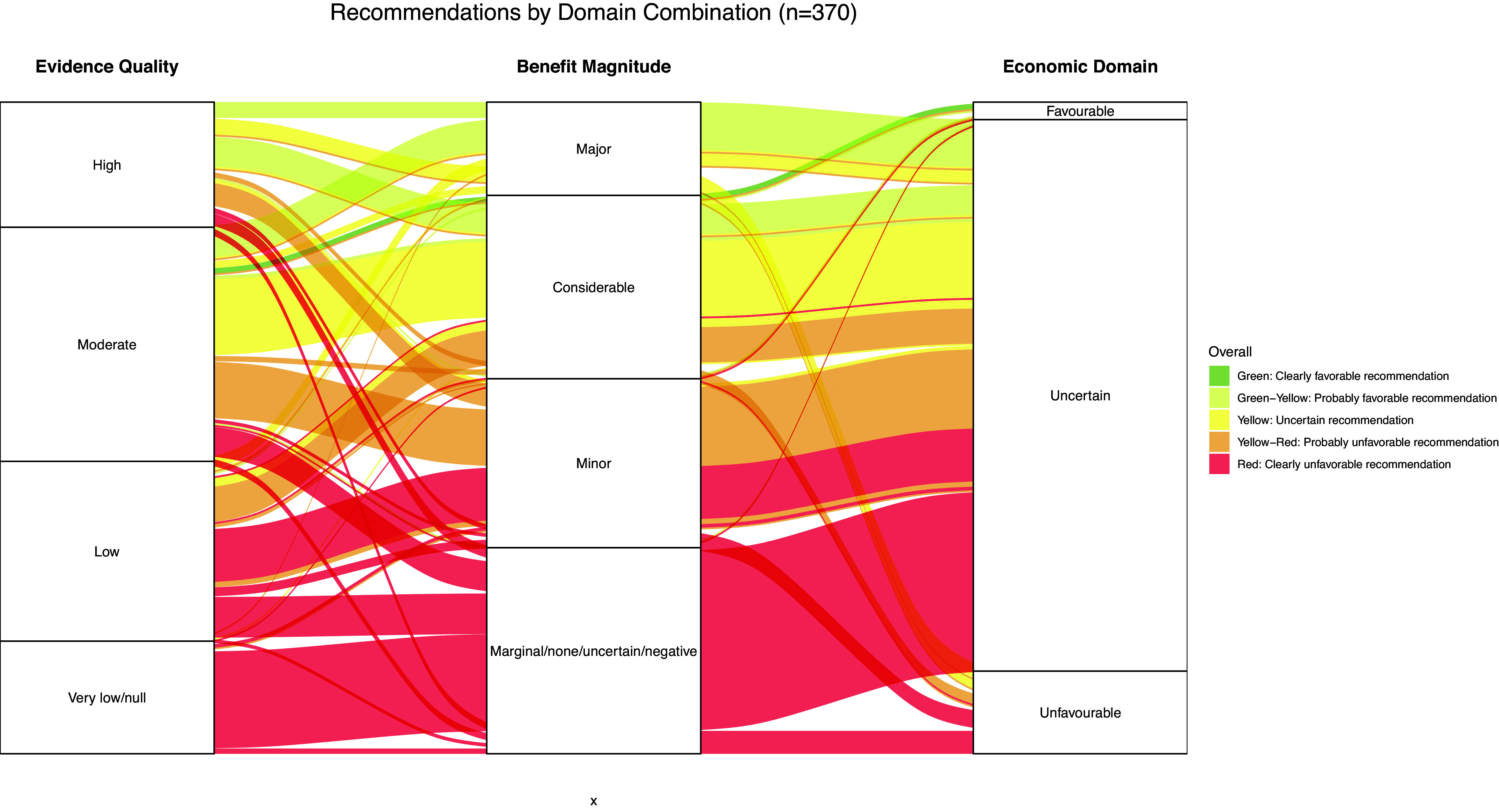
Recommendations by classification of evidence quality, expected benefit, and economic domain. *This figure displays the combinations of three core decision-making criteria used in HTA reports: the quality of the evidence (left column), the magnitude of expected benefit (middle column), and the economic domain (right column). Each horizontal line across these columns represents a specific report, with the positions of the marks indicating the level assessed for each criterion. Each line’s color reflects the recommendation’s overall favorability, ranging from green (clearly favorable) to red (clearly unfavorable). The figure illustrates the variability in how different combinations of evidence, benefits, and economic considerations lead to final recommendations. Three reports were excluded due to lack of data, while two studies were omitted due to changes in the matrix assessment in the last months, where the economic domain was not evaluated due to very low/null evidence. Green: fully in favor; green-yellow: moderately in favor; yellow: uncertain; yellow-red: moderately against; red: strongly against.*

**Figure 3.**
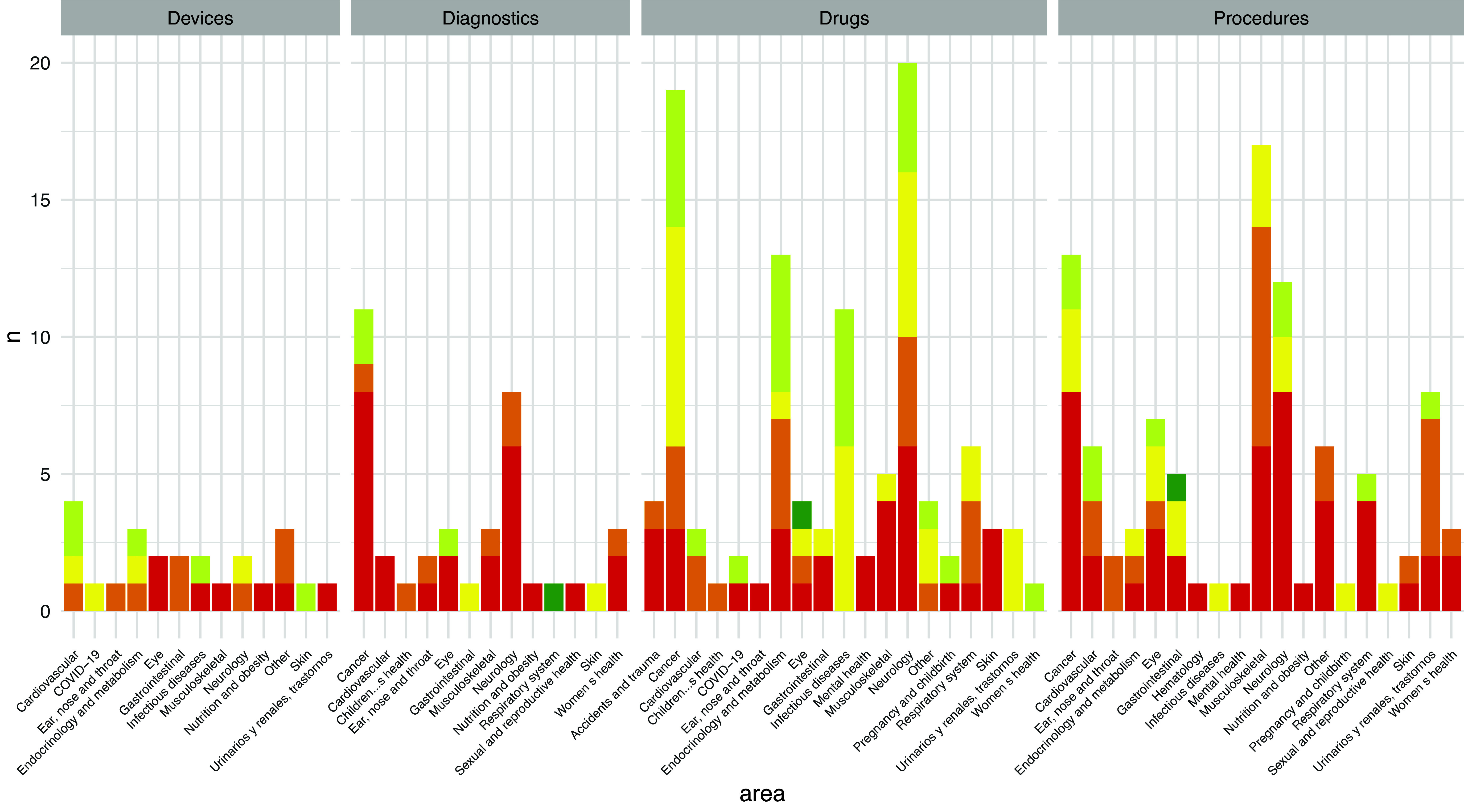
Level of HTA recommendations according to the healthcare area and type of technology assessed.

Of the 375 recommendations, 66.3 percent were moderately or totally unfavorable (22.1 percent and 44.3 percent, respectively) and only 0.8 percent were totally favorable. Figure 2 shows how the recommendations are distributed based on the three criteria considered (evidence quality, benefit, and economics) and Figure 3 shows the recommendation/color of the same according to type of technology and area. Of the 166 recommendations that were *totally against* coverage (44 percent of the total): 55 originate from a combination of very low-quality evidence, marginal/no/uncertain benefit, and uncertain economics; 23 showed the same pattern, but with low-quality evidence, 17 had moderate quality evidence; and 29 presented a combination of low evidence quality, minor benefit, and uncertain economics. Of the 83 *moderately against* recommendations (22.2 percent of the total): 30 come from a moderate evidence quality/minor benefit combination, 20 low evidence quality/considerable benefit, and 13 high evidence quality/minor benefit, all with uncertain economics. Of the 77 *uncertain* recommendations (20.5 percent of the total), the pattern most frequently found was that of moderate evidence quality, with considerable benefit and uncertain economics. Of the 49 *moderately favorable* recommendations, 17 showed a combination of very low-quality evidence with minor benefit, and 18 moderate quality evidence with major benefit, both with uncertain economics. Only three *totally favorable* recommendations were observed, with a combination of moderate quality evidence, with considerable benefit and favorable economics (Figure 1 and Annex 2).

Figure 3 shows the distribution of the assessments with their respective recommendation levels (for the main recommendation when more than one was available) according to the condition areas and technology type. The figure shows that most drug assessments were conducted in the areas of neurology and cancer, followed by endocrinology and infectious diseases, with recommendations ranging from *uncertain* to *moderately favorable.* In the case of procedures, though the areas of neurology and cancer were frequently evaluated, most assessments focused on musculoskeletal conditions, with recommendations most often between *totally against* and *moderately against.* For diagnostic technologies, the most assessed areas were cancer and neurology, with a preponderance of red recommendations.

## Discussion

This article presents the development and implementation experience of a value framework (VF) created by the Institute for Clinical Effectiveness and Health Policy in Argentina. Designed to support rapid HTAs, the VF proved versatile in addressing requests from diverse funders and for different types of technologies. Its implementation was feasible across various clinical areas, and the assessments were completed within 4 to 8 weeks, demonstrating the framework’s responsiveness and flexibility in real-world settings.

The VF was developed in response to the increasing demand for timely evaluations in contexts with limited resources and data. Adaptive HTA (aHTA) has emerged as a pragmatic solution in such scenarios, aiming to balance the need for speed with acceptable trade-offs in methodological rigor. A recent scoping review identified 20 countries and one HTA network using aHTA methods; IECS was recognized among the centers applying this approach in Argentina. While aHTA methods are faster and more efficient, they have been criticized for lacking transparency and standardized processes – highlighting the importance of structured, context-sensitive models like the VF described here (12;13).

A key feature of the VF is its explicit incorporation of three core domains commonly used in HTA: clinical benefit, quality of evidence, and economic impact. These are translated into a structured weighting system that leads to a coverage recommendation, offering decision makers actionable guidance while preserving methodological transparency. However, only a small proportion of the assessments (seven reports) met the criteria for both high-quality evidence and favorable economic impact. In contrast, 166 assessments identified a lack of adequate evidence – reflecting a common challenge in LMICs where decisions often rely on imperfect or incomplete information.

One methodological challenge was the application of the net benefit criterion, adapted from the IQWiG methodology (9). Although it provided a conceptual basis, the method did not offer a formal mechanism for aggregating benefits across multiple outcomes or for comparing benefits and harms. The process relied on structured deliberation by researchers, starting with the most influential outcome and incorporating contextual adjustments. This deliberation, while essential, introduces variability and may affect the reproducibility of results over time. Similar challenges arise when ranking the overall quality of evidence, especially when outcomes differ in importance or certainty (11;14).

The economic domain emerged as the most difficult to operationalize. Traditional cost-effectiveness models were unsuitable given the lack of defined thresholds in Argentina and the time constraints of rapid HTAs. A tailored method was developed to estimate economic impact using international criteria (e.g., from Chile and England), adjusted for local purchasing power, and informed by contextual elements such as target population size, net benefit, and decisions in other health systems. Still, most assessments resulted in an “uncertain” classification for this domain – underscoring the need to refine economic evaluation approaches for use in aHTAs. Several alternatives are under exploration, such as those from the Inter-American Development Bank and India’s model for cost-effectiveness without full evaluations, though their adoption remains limited (15–17).

The application of the VF to specific technologies also presented challenges. Diagnostic technologies require assessing both accuracy and clinical implications, which often demand additional time and tailored criteria. Although IECS developed a dedicated VF and operational manual for diagnostics, aligning it with the general VF proved complex (18;19). Likewise, “me-too” drugs – structurally similar compounds with marginal differences – pose difficulties for value assessments, as they may be penalized under the matrix despite potential cost advantages, if their net benefit appears null (20).

A primary limitation of this work is that the assessments generated recommendations, but not final coverage or policy decisions. Although the VF was designed to inform decision making, the ultimate adoption of a technology depends on payers and health authorities. Therefore, this study does not evaluate the real-world policy impact of the recommendations. Additionally, while the framework incorporates structured criteria, the reliance on deliberation may introduce subjectivity and inconsistencies across time or evaluators.

Nonetheless, many of these challenges are not unique to this VF. They reflect broader debates within HTA, particularly in resource-constrained environments. The IECS framework continues to evolve through ongoing methodological refinement. Despite its limitations, it remains a feasible, transparent, and adaptable tool that supports timely and structured HTA production across a wide range of technologies and decision contexts.

## Supporting information

Alcaraz et al. supplementary material 1 Alcaraz et al. supplementary material

Alcaraz et al. supplementary material 2 Alcaraz et al. supplementary material

## Supplementary material

The supplementary material for this article can be found at http://doi.org/10.1017/S0266462325100160.
