# Supplementary material for: Development and implementation of a value framework for rapid health technology assessment reports: enhancing evidence-informed decision making in resource-constrained settings
Source: Int J Technol Assess Health Care. 2025 Jul 21;41(1):e58. doi: 10.1017/S0266462325100160 (PMC12390745; doi:10.1017/S0266462325100160)
Supplement: Alcaraz et al. supplementary material 1 — Alcaraz et al. supplementary material [file S0266462325100160sup001.pdf]

Annex 1 – Colour Scale Table for Value Judgement Criteria

EVIDENCE QUALITY	BENEFIT	ECONOMIC IMPACT	COLOR
High	Major	Reasonable/Low	1
High	Considerable	Reasonable/Low	1
High	Minor	Reasonable/Low	5
High	Marginal / Null / Negative	Reasonable/Low	2
Moderate	Major	Reasonable/Low	1
Moderate	Considerable	Reasonable/Low	1
Moderate	Minor	Reasonable/Low	5
Moderate	Marginal / Null / Negative	Reasonable/Low	2
Low	Major	Reasonable/Low	5
Low	Considerable	Reasonable/Low	5
Low	Minor	Reasonable/Low	2
Low	Marginal / Null / Negative	Reasonable/Low	2
Very Low/None	Major	Reasonable/Low	4
Very Low/None	Considerable	Reasonable/Low	4
Very Low/None	Minor	Reasonable/Low	2
Very Low/None	Marginal / Null / Negative	Reasonable/Low	2
High	Major	Uncertain	5
High	Considerable	Uncertain	5
High	Minor	Uncertain	4
High	Marginal / Null / Negative	Uncertain	2
Moderate	Major	Uncertain	5
Moderate	Considerable	Uncertain	3
Moderate	Minor	Uncertain	4
Moderate	Marginal / Null / Negative	Uncertain	2
Low	Major	Uncertain	3
Low	Considerable	Uncertain	4
Low	Minor	Uncertain	2
Low	Marginal / Null / Negative	Uncertain	2
Very Low/None	Major	Uncertain	4
Very Low/None	Considerable	Uncertain	4
Very Low/None	Minor	Uncertain	2
Very Low/None	Marginal / Null / Negative	Uncertain	2
High	Major	Unreasonable	3
High	Considerable	Unreasonable	4
High	Minor	Unreasonable	2
High	Marginal / Null / Negative	Unreasonable	2
Moderate	Major	Unreasonable	3
Moderate	Considerable	Unreasonable	4
Moderate	Minor	Unreasonable	2
Moderate	Marginal / Null / Negative	Unreasonable	2
Low	Major	Unreasonable	4
Low	Considerable	Unreasonable	2
Low	Minor	Unreasonable	2
Low	Marginal / Null / Negative	Unreasonable	2
Very Low/None	Major	Unreasonable	2
Very Low/None	Considerable	Unreasonable	2
Very Low/None	Minor	Unreasonable	2
Very Low/None	Marginal / Null / Negative	Unreasonable	2
