# Supplementary material for: Development and implementation of a value framework for rapid health technology assessment reports: enhancing evidence-informed decision making in resource-constrained settings
Source: Int J Technol Assess Health Care. 2025 Jul 21;41(1):e58. doi: 10.1017/S0266462325100160 (PMC12390745; doi:10.1017/S0266462325100160)
Supplement: Alcaraz et al. supplementary material 2 — Alcaraz et al. supplementary material [file S0266462325100160sup002.pdf]

Annex 2 – Dataset of 375 HTA Recommendations by Evidence, Benefit, and Economics

Overall	Evidence	Benefit	Economics	n
YELLOW	High	Considerable	Uncertain	1
YELLOW	High	Major	Unfavorable	9
YELLOW	High	Minor	Uncertain	2
YELLOW	Low	Considerable	Uncertain	5
YELLOW	Low	Major	Uncertain	7
YELLOW	Low	Major	Unfavorable	1
YELLOW	Moderate	Considerable	Uncertain	43
YELLOW	Moderate	Major	Uncertain	1
YELLOW	Moderate	Major	Unfavorable	4
YELLOW	Very low/nul	Considerable	Uncertain	1
YELLOW & RED	High	Considerable	Uncertain	1
YELLOW & RED	High	Considerable	Unfavorable	3
YELLOW & RED	High	Major	Unfavorable	1
YELLOW & RED	High	Minor	Uncertain	13
YELLOW & RED	Low	Considerable	Uncertain	20
YELLOW & RED	Low	Considerable	Unfavorable	1
YELLOW & RED	Low	Major	Unfavorable	1
YELLOW & RED	Low	Minor	Favorable	1
YELLOW & RED	Low	Minor	Uncertain	3
YELLOW & RED	Moderate	Considerable	Unfavorable	3
YELLOW & RED	Moderate	Considerable	Favorable	1
YELLOW & RED	Moderate	Major	Uncertain	1
YELLOW & RED	Moderate	Minor	Uncertain	32
YELLOW & RED	Very low/nul	Major	Uncertain	1
YELLOW & RED	Very low/nul	Minor	Uncertain	1
RED	High	Marginal/None/Uncertain	Uncertain	7
RED	High	Marginal/None/Uncertain	Unfavorable	4
RED	High	Minor	Unfavorable	3
RED	Low	Considerable	Uncertain	1
RED	Low	Considerable	Unfavorable	1
RED	Low	Marginal/None/Uncertain	Uncertain	23
RED	Low	Marginal/None/Uncertain	Unfavorable	2
RED	Low	Minor	Uncertain	30
RED	Low	Minor	Unfavorable	5
RED	Moderate	Marginal/None/Uncertain	Favorable	1
RED	Moderate	Marginal/None/Uncertain	Uncertain	17
RED	Moderate	Marginal/None/Uncertain	Unfavorable	4
RED	Moderate	Minor	Unfavorable	2
RED	Very low/nul	Marginal/None/Uncertain	Uncertain	55
RED	Very low/nul	Marginal/None/Uncertain	Unfavorable	3
RED	Very low/nul	Minor	Uncertain	2
RED	Very low/nul	Minor	Favorable	1
GREEN	Moderate	Considerable	Favorable	3
GREEN & YELLOW	High	Considerable	Uncertain	17
GREEN & YELLOW	High	Major	Uncertain	9
GREEN & YELLOW	High	Minor	Favorable	1
GREEN & YELLOW	Low	Considerable	Favorable	1
GREEN & YELLOW	Moderate	Considerable	Uncertain	2
GREEN & YELLOW	Moderate	Marginal/None/Uncertain	Favorable	1
GREEN & YELLOW	Moderate	Major	Uncertain	18
